# C. J. M. Langenbeck's Anatomical Plates

**Published:** 1842-10

**Authors:** 


ART. XVI.—CONRADI JOANNIS MARTINI LANGENBECK, *Icones Anatomicæ.—Gottingæ*, 1842. Sumptibus Auctoris.

C. J. M. LANGENBECK'S *Anatomical Plates.—Göttingen*, 1842.

THESE plates have been drawn and engraved from dissections made by the celebrated Langenbeck of Göttingen, or under his direction. They are divided into eight *fasciculi*, three of which are occupied with the anatomy of the nervous system, and two with that of the vascular system. The viscera, muscles, and bones and ligaments occupy the remaining three; together with three plates representing the anatomy of the operations performed for ligature of the arteries. These last, and the plates exhibiting the anatomy of the muscles and bones and ligaments, are lithographs.

We have looked over these anatomical plates and can testify to their general accuracy. Having been engraved by different hands, they are, however, of unequal merit. The three neurological *fasciculi* contain engravings which, for novelty, beauty, and accuracy, are unrivalled. The drawing, engraving, printing, and paper are all unexceptionably good. There are sixty-four plates in these three fasciculi, containing nearly 130 figures. They exhibit the nervous system in all its relations, in all its ramifications, and in every possible point of view. Sixty-two of these figures are devoted solely to the anatomy of the cerebrum and cerebellum, and show several sections of these organs quite new to us. Those who desire to possess a thorough knowledge of the anatomy of the encephalon and of the complicated relations of its various parts, will find these plates invaluable. The successive course of the fibrils through the cerebral ganglia and their distribution in the cerebellum are admirably delineated. These neurological plates could scarcely be considered dear at half the price of the whole set. The anatomy of the blood-vessels is shown in a rather stiff style, and some of the engravers have not possessed the skill of the artist employed on the neurological drawings. However, they are generally accurate, and that is no slight merit. The engravings representing the anatomy of the eye and ear deserve special mention; and those of the heart are tolerably good.

We cannot speak favorably of the printing of the lithographs. Several of the prints in our possession have been struck off in a very slovenly and unworkmanlike manner, and the paper is of inferior quality. Nor can we compliment Messrs. "Siedentopf und Sohn" of Göttingen on their skill as copper-plate printers, or on their taste. It is surprising that Professor Langenbeck should have allowed those persons to disfigure his plates by scrawling their own euphonious names on them in the way they have.

The plates correspond in size to those of Tiedemann's. They would certainly have been more convenient if got up in a more portable form. As a whole, they will add to Professor Langenbeck's high reputation and cannot fail to be valued by both student and practitioner.

